# The role of tungsten oxide in Er^3+^-doped bismuth-germanate glasses for optical amplification in L-band

**DOI:** 10.1038/s41598-023-35995-8

**Published:** 2023-05-31

**Authors:** Hüseyin Can Çamiçi, Théo Guérineau, V. A. G. Rivera, Rodrigo Ferreira Falci, Sophie LaRochelle, Younès Messaddeq

**Affiliations:** Centre for Optics, Photonics and Laser (COPL), Université Laval, 2375 rue de la Terrasse, Quebec, QC Canada

**Keywords:** Optical materials and structures, Fibre optics and optical communications, Mid-infrared photonics

## Abstract

A series of novel Er^3+^-doped bismuth-germanate glasses containing different tungsten concentrations with a molar composition of 97.5[(75 − x)GeO_2_–25Bi_2_O_3_–(x)WO_3_]–2Sb_2_O_3_–0.5Er_2_O_3_ (x = 5, 10, 15, 20, and 25 mol%) were fabricated. Their thermal properties are measured by differential scanning calorimetry. A structural investigation by Raman spectroscopy suggested that changes occurred in the glass network by WO_3_ incorporation. By laser excitation at 980 nm, a strong emission from Er^3+^ ions at 1532 nm is observed, while the WO_3_ addition caused changes in the emission spectra. The emission cross-section spectra of Er^3+^ are calculated by both McCumber and Füchtbauer–Ladenburg theories and their comparison showed these theories yielded slightly different results, but in both cases, the calculations showed that a gain signal in L-band can be achieved when 30% of the Er^3+^ ions are at the excited state. This study proves that the Er^3+^-doped bismuth-germanate glasses are suitable for optical fiber amplifier applications operating at C- and L-band.

## Introduction

Rare-earth-doped fibers are attracting a lot of attention for their appropriate properties to amplify optical signals. Silica-based Er^3+^-doped fibers have been used in optical communication systems for many years. However, Er^3+^ ions have low solubility and narrow fluorescence spectrum in silica, which results in Er^3+^-doped silica fibers working relatively inefficiently in the L-band^1,2^. The high demand for information transmission on the communication networks has made it urgent to employ erbium-doped fiber amplifiers (EDFA) in the L-band^3^. Wavelength division multiplexing (WDM) systems are widely used in EDFAs and improving the capacity of WDMs by broadening their operating wavelength range would increase the efficiency of EDFAs in return^4^. There is a lot of research devoted to achieving EDFAs with broad gain bandwidth and high gain in new glass host materials such as silicate, germanate, and tellurite glasses^5–7^.

Germanate-based heavy metal oxide (HMO) glasses and fibers, especially lead-germanate, have been extensively studied and they are shown to be efficient in amplifying optical signals in the L-band^8–10^. Germanate-based HMO glasses yield medium and long-range structural disorder as a characteristic of a vitreous system that improves the thermal stability of the glass and solubility of rare-earth ions (REI), giving great potential for applications in optical communications^6,11–13^. However, due to the actual worldwide regulation of lead, bismuth-germanate glasses have been proposed as an alternative glass matrix to be deployed in optical amplifiers for their appropriate properties. Thanks to their high refractive index (~ 2), low phonon energy (~ 700 cm^−1^), high REI solubility, high mid-infrared (MIR) transmission up to 5 µm, high thermal stability, good mechanical strength and good chemical durability, bismuth-germanate glasses are great candidates for optical devices and photonic applications^14–18^. Generally, in a glass doped with REIs, a high refractive index increases the spontaneous emission probability, while in the meantime, a low phonon energy enhances the radiative relaxation rate from an excited energy state to a lower energy state. Hence, the resulting combination of these two effects would help broaden the spectral distribution of fluorescence from REIs^19–21^.

Some researchers have studied the influence of the glass optical basicity on the spectral distribution of the Er^3+^ emission spectrum previously^22–24^. For each glass system under study, Tanabe et al*.* have evidenced that low glass optical basicity decreases the bond covalency between the Er^3+^ ions and glass matrix ions, allowing Er^3+^ ions to have a broader emission spectrum^19^. Additionally, it has been revealed that low optical basicity is achieved by incorporating cations with high electronegativity values into the glass matrix^19^. Bismuth-germanate glasses consist mainly of germanium and bismuth cations with electronegativities of 2.01 and 2.02, respectively, which are higher than that of silicon, 1.9, and other cations that silica glasses may include.

In this work, the electronegativity of erbium-doped bismuth-germanate glasses is increased by substituting germanium atoms with tungsten atoms with an electronegativity of 2.36, leading to a lower optical basicity of the glasses and a broader fluorescence spectrum. These glasses with varying tungsten contents are systematically studied in terms of thermal, structural, and spectroscopic properties, and lifetime measurements. Besides, gain bandwidth and gain properties in the samples are calculated.

## Results and discussion

The thermal and physical properties of prepared glasses are given in Table 1.

**Table 1:** T_g_, T_x_, ΔT, density, Er^3+^ ion concentration and the polarizability at 1538 nm values of the glass samples.

Sample	T_g_ (± 3 °C)	T_x_ (± 3 °C)	ΔT (± 6 °C) (T_x_–T_g_)	Glass Density (± 0.01 g/cm^3^)	Er^3+^ ion concentration ($\times$ 10^20^ ions/cm^3^)	Polarizability at 1538 nm ($\times$ 10^–24^)
5W	493	706	213	6.04	1.78	6.44
10W	495	607	112	6.16	1.76	6.53
15W	501	584	83	6.35	1.74	6.83
20W	496	582	86	6.51	1.73	6.97
25W	497	580	83	6.63	1.72	7.14

The T_g_ of the glasses maintain similar values at around 496 ± 5 °C while there is a decrease in T_x_, hence, the thermal stability of these samples weakens when the WO_3_ nominal content is greater than 10 mol%. The T_g_ and T_x_ values and the difference between them, ΔT, are shown in Table 1. The ΔT is described as the thermal stability of the glass and regarded as a measure of the crystallization tendency of the glass. Generally, the ΔT is desired to be greater than 100 °C for fiber fabrication. Therefore, the samples 5W and 10W seem to have appropriate thermal properties to be drawn into the fiber. The influence of the increase in WO_3_ content over the glass density and Er^3+^ ion concentration in the glass is also shown in Table 1. As the WO_3_ content increases from 5 to 25 mol%, the glass density rises from 6.04 to 6.63 g/cm^3^, while the Er^3+^ ion concentration decreases slightly from 1.78 $\times$ 10^20^ to 1.72 $\times$ 10^20^ ions/cm^3^. Additionally, the glass polarizabilities of the glasses at 1538 nm are calculated from the Clausius–Mossotti relation $P=\frac{3\times ({n}^{2}-1)\times M}{4\times ({n}^{2}+2)\times \rho \times \pi \times N}$ where $P$ is the glass polarizability, $n$ is the glass refractive index at 1538 nm, $M$ is the molecular weight of the glass, $\rho$ is the glass density and $N$ is Avogadro’s number^25–27^. Table 1 shows that the glass polarizability escalates with the WO_3_ content and, thus, with the glass density.

**Figure 1:**
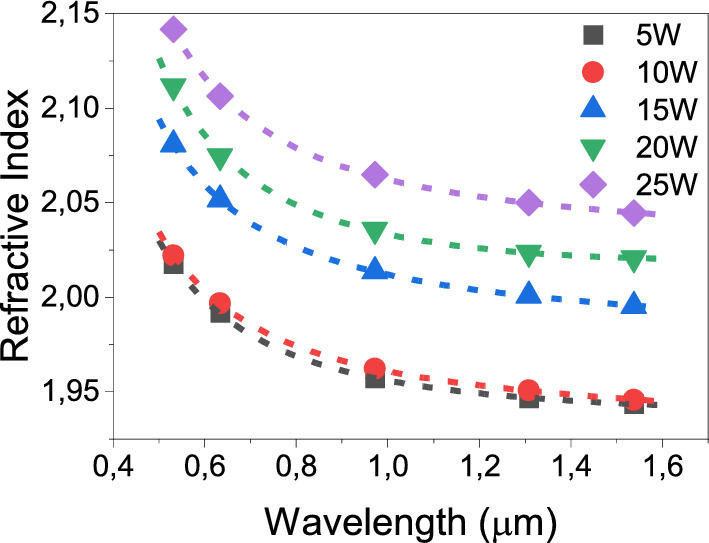
Refractive indices of the glasses measured at 532, 633, 972, 1308, and 1538 nm by prism coupling technique.

The refractive indices of the glass samples with respect to the WO_3_ content as a function of the wavelength are shown in Fig. 1. The refractive index increases from around 1.95 to 2.05 at 1538 nm as the WO_3_ content rises from 5 to 25 mol%. A decrease in the refractive indices at longer wavelengths is also exhibited.

The refractive index measurements for each sample in Fig. 1 are fitted with the Sellmeier equation^28^ ${n\left(\lambda \right)}^{2}=1+(\frac{{B}_{1}\times {\lambda }^{2}}{{\lambda }^{2}-{C}_{1}}+\frac{{B}_{2}\times {\lambda }^{2}}{{\lambda }^{2}-{C}_{2}})$, where $n$ is the refractive index of the samples, $\lambda$ is the wavelength, ${B}_{1}$, ${B}_{2}$, ${C}_{1}$ and ${C}_{2}$ are the dimensionless Sellmeier equation coefficients, which can be found in Supplementary Table S1. The fittings made by the Sellmeier constants agree with the refractive index values in Fig. 1. The ${B}_{1}$ parameter is associated with the network disorder in glasses and an increase in ${B}_{1}$ is measured, which might indicate that the introduction of a greater proportion of W–O bonds increases the network disorder in bismuth-germanate glasses. The negative values of Sellmeier’s coefficients have already been reported for a germanate matrix^13,29^. The regression curves for all samples are obtained with an R^2^ value higher than 0.99.

**Figure 2:**
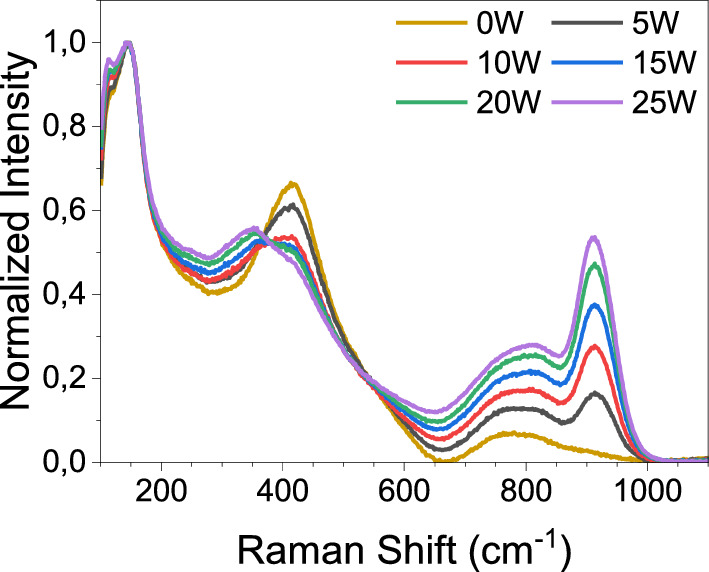
Normalized raw data Raman spectra of glass samples excited by 785 nm laser excitation.

Figure 2 shows the normalized Raman spectra of the glass samples. For this measurement, a glass with 0 mol% WO_3_ is prepared to observe the effect of WO_3_ addition. Some changes take place in the glass structure as the WO_3_ content increases. The emergence of a band at 913 cm^−1^ is attributed to symmetric stretching vibrations of terminal W–O bonds (W–O^−^ or W=O bonded species)^30,31^. The intensity increase in this band may be inferred as the addition of a higher amount of WO_3_ into the glass structure. Additionally, it can be seen that the intensity of the band at 413 cm^−1^ decreases while the one at 352 cm^−1^ improves as the WO_3_ content of the glasses increases. The band at 413 cm^−1^ is attributed to the symmetric vibrations of Ge–O bonds and the band at 352 cm^−1^ is associated with bending vibrations of W–O terminal bonds in WO_6_ octahedra^32,33^. The former band shrinks, whereas the latter grows as the WO_3_ content of the glass becomes greater. Due to GeO_2_ being the host element, the bridging oxygens (BO) are formed by O–Ge–O or Ge–O, but, when the WO_3_ content increases, the amount of BO decreases and non-bridging-oxygens (NBO) appears and increases with the WO_3_ content. The opposite change in the bands at 413 cm^−1^ and 352 cm^−1^ might be inferred as the breaking of Ge–O bonds and creation of W–O bonds. The increase in the NBOs quantity would increase the glass polarizability; thus, increase the refractive index as well^34,35^. This effect of the NBOs might confirm the glass density and polarizability increase in Tables 1 and 2. Moreover, the band at 800 cm^−1^ is due to the antisymmetric stretching vibrations of Ge–O–Ge bonds^36^. The appearance of a new band at 830 cm^−1^ is claimed to be due to W–O–W bonds^31^. Finally, the band at 145 cm^−1^ is attributed to the vibrations involving bismuth^37^.

**Figure 3:**
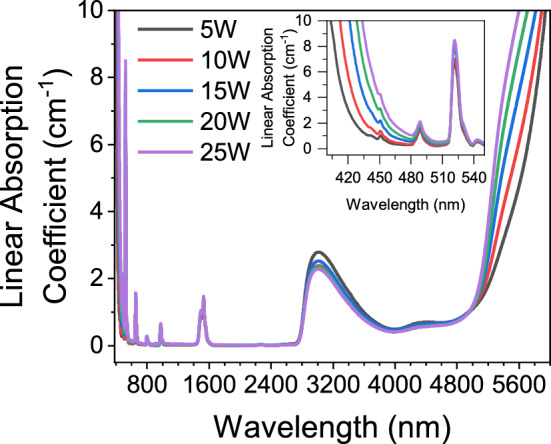
Linear absorption coefficient spectra of the samples between 380 and 6000 nm. The inset shows the evolution of the UV absorption edge by the tungsten addition.

Linear absorption coefficient spectra of the glasses are shown in Fig. 3. The absorption peaks at 451, 488, 521, 543, 653, 797, 977, and 1531 nm shown in Fig. 3 are due to the 4f–4f transitions of Er^3+^ ions, which are ^4^F_3/2_ + ^4^F_5/2_, ^4^F_7/2_, ^2^H_11/2_, ^4^S_3/2_, ^4^F_9/2_, ^4^I_9/2_, ^4^I_11/2_, and ^4^I_13/2_, respectively. From the inset of Fig. 3, it is shown that the UV absorption edge of the glasses exhibit a red shift of 30 nm because of the addition of tungsten since the tungsten atoms absorb the blue light^38^. Additionally, Fig. 3. reveals an absorption band at around 3000 nm, which is caused by the OH^−^ present in the glasses. Furthermore, Fig. 3 shows that the glass samples reach their MIR transmission limit near 6000 nm. With the increase in the WO_3_ content, this MIR limit experience a blue shift which might be interpreted as that phonon energies of the glasses increase^39^.

**Figure 4:**
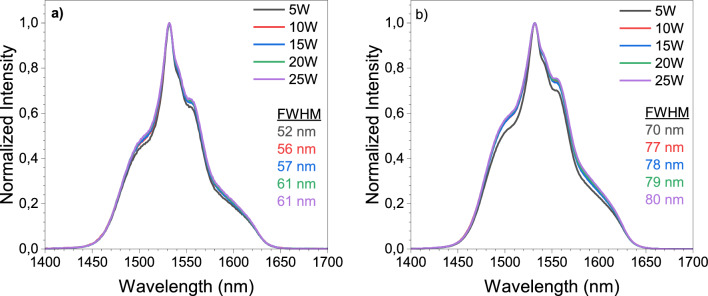
Normalized Er^3+^ emission intensity spectra for excitation by a 980 nm LD at (**a**) 200 mW and (**b**) 250 mW.

The normalized Er^3+^ emission spectra of the prepared glasses excited at 200 and 250 mW laser power are presented in Fig. 4. These spectra in Fig. 4a,b express the emission broadening obtained by increasing the number of tungsten atoms in the glass matrix. By enhancing the number of excitation sites, the tungsten addition might have helped more electrons to be promoted on the different energy sublevels (due to the Stark effect) of the ^4^I_13/2_ of the Er^3+^ ions. The resulting emission spectra broadening is inhomogeneous and depends on the crystal field in different sites, hence, different transition probabilities can be produced with the increase in the tungsten content in the glass. Full width at half maximum (FWHM) values show an increase from 52 to 61 nm for 200 mW and from 70 to 80 nm for 250 mW pumping power. Additionally, the FWHM values for the same pumping power increase because of the higher amount of tungsten, which has a high electronegativity and low optical basicity, decreasing the bond covalency between the Er^3+^ and oxide ions which causes the Er^3+^ emission spectrum to broaden^4^. Figure 4a,b also reveal that the emission spectra broaden when the samples are excited by a higher laser power, which is not related to the sample reabsorption.

In contrast to the other oxide glass precursors, germanium oxide is not a conditional glass former. Nevertheless, no metallic germanium is present in our glasses, as it is only present in oxide form. Hence, no luminescence from such germanium form is observed. Therefore, the Er^3+^ emission band in the NIR is unaffected by germanium.

It is important to mention that the energy transfer from Bi^3+^ ions to Er^3+^ ions was discussed in the literature before^40,41^. However, this transfer takes place when the Bi^3+^ ions are excited in the UV range at around 340 nm. Here, the glasses were excited with a 980 nm and such excitation wavelength is outside of Bi^3+^ ions energy levels. Thus, an energy transfer process from Bi^3+^ ions to Er^3+^ ions is not expected. This is confirmed in the emission spectra, Fig. 4.

The Er^3+^ lifetime values were 4.06, 4.02, 3.79, 3.68 and 3.62 ms for the samples 5W, 10W, 15W, 20W, and 25W, respectively. In all samples, one exponential decay is observed. The decay curves are displayed in the inset of Supplementary Fig. S2 of Supplementary Material. Additionally, it is demonstrated that the radiative lifetime decreases with the increasing tungsten atoms in the glass network, which increases the refractive index of the glasses as well (Fig. 1)^42^. Also, the intensity of emission is related to the radiative transition probabilities and it is inversely related with the lifetime which are also true for these samples^43^.

**Figure 5:**
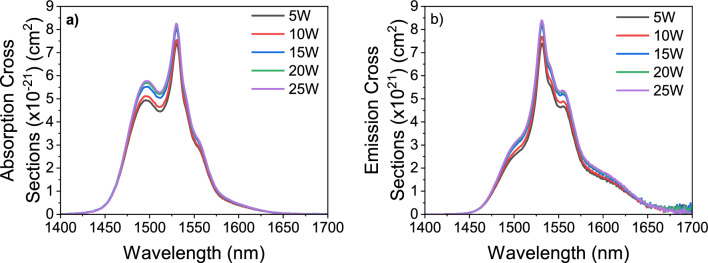
(**a**) Absorption (**b**) Emission cross-sections of the Er^3+^ ions by the McCumber theory.

By using the linear absorption coefficients of Er^3+^ ions, absorption and emission cross-section spectra are calculated by the McCumber (MC) theory which are shown in Fig. 5. This theory uses the linear absorption coefficients of Er^3+^ ions for the emission cross-section approximation as in the formula: ${\sigma }_{ems}\left(v\right)={\sigma }_{abs}\left(v\right)\mathrm{exp }(\frac{hv-{E}_{0}}{{k}_{B}T})$ where ${\sigma }_{ems}\left(v\right)$ is the emission cross-section, ${\sigma }_{abs}\left(v\right)$ is the absorption cross-section, $h$ is the Planck constant, $v$ is the optical frequency, ${k}_{B}$ is the Boltzmann constant and $T$ is temperature^44^. ${E}_{0}$ can be calculated from the energies of the single Stark levels. Figure 5 shows that the absorption and emission cross-sections of the Er^3+^ ions improved with increasing WO_3_ content from 5 to 25 mol%. In addition, the emission spectra of Er^3+^ ions broaden with the incorporation of more WO_3_.

**Table 2:** Emission cross-sections and Judd–Ofelt intensity parameters Ω_λ_ (λ = 2, 4, 6) of Er^3+^ in different glass hosts.

Glass composition	${\sigma }_{ems}$ (10^–21^ cm^2^)	Ω_2_ (10^–20^ cm^2^)	Ω_4_ (10^–20^ cm^2^)	Ω_6_ (10^–20^ cm^2^)	References
5W	7.4	6.89	1.94	1	This work
10W	7.7	8.11	2.04	1.04	This work
GeO_2_–Bi_2_O_3_	7	4.5	1.6	0.7	^48^
GeO_2_–Bi_2_O_3_–PbO	7	4.1	1.6	0.2	^9^
GeO_2_–PbO	6	4.1	1.6	0.5	^9^
Silicate	4.5	5	2.1	1	^49^
Tellurite	7.5	4.98	1.25	1.43	^50^
ZBLAN	5.8	2.9	1.8	1	^51,52^

The maximum values obtained from the emission cross-sections are 7.4 $\times$ 10^–21^ cm^2^ and 7.7 $\times$ 10^–21^ cm^2^ for 5W and 10W, respectively. These values and others from different glass systems are shown in Table 2. The emission cross-sections of 5W and 10W are similar to the other bismuth-germanate, lead-germanate and tellurite glasses, whereas they are higher than silicate glasses. A similar comparison can be made from Table 10 of Digonnet’s work^45^. To provide high gain to fabricate fiber amplifier, the emission cross-section is required to be as high as possible; therefore, bismuth-germanate glasses are a good candidate for such applications. Furthermore, Judd–Ofelt intensity parameters are calculated from the Judd–Ofelt theory^46,47^ and compared with other glass families in Table 2 as well. The Ω_2_ parameter describes the covalency between the REIs and the surrounding oxygen ions. The Ω_2_ values of 5W and 10W are greater than other germanate glasses, silicates and tellurites, which implies that these samples have much weaker covalency than other glasses which is obtained by tungsten addition as explained before. The Ω_4_ and Ω_6_ parameters exhibit the covalency of the glass matrix. Moreover, the Ω_6_ is shown to be the most important Judd–Ofelt parameter to increase the line strength of the electric dipole and the spontaneous emission probability^19^. It can be seen that the Ω_6_ is improved by increasing the WO_3_ content from 5 to 10 mol%. Therefore, samples 5W and 10W are capable of yielding a broader emission bandwidth than lead-germanate glasses.

**Figure 6:**
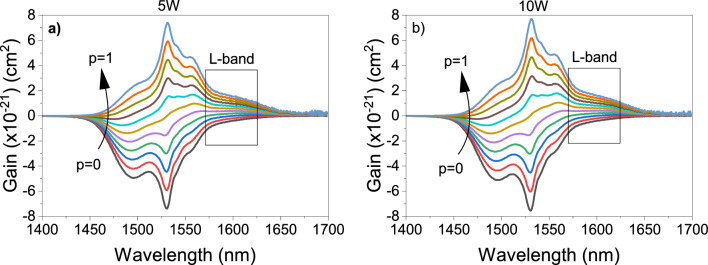
Gain spectra by McCumber theory of the samples with (**a**) 5 mol% WO_3_ and (**b**) 10 mol% WO_3_.

After that, the gain spectra are calculated from the results of the MC theory, which the results for the samples 5W and 10W are shown in Fig. 6 since they are the candidates for fiber fabrication due to their ΔT being greater than 100 °C. However, the results of the other samples are quite similar. The gain spectra are calculated from $Gain=[{p\times \sigma }_{ems}\left(v\right)-(1-p)\times {\sigma }_{abs}\left(v\right)]$ where $p$ is the percentage of Er^3+^ ions at the excited level (the population inversion ratio between the ^4^I_13/2_ and ^4^I_15/2_ energy levels). From Fig. 6a,b, it is exhibited that at least 30% of the Er^3+^ ions should be at the excited state level to obtain gain in the L-band. For greater population inversion, gain becomes positive for the L-band spreading from 1565 to 1640 nm.

**Figure 7:**
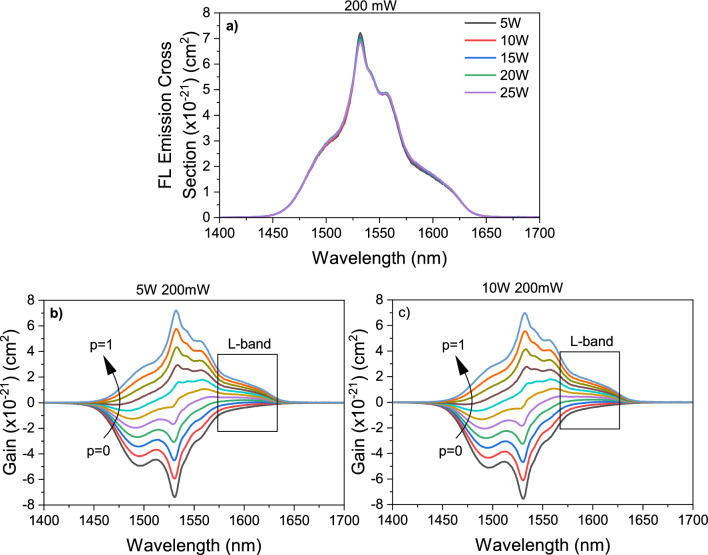
(**a**) Emission cross-sections of glasses by the Füchtbauer–Ladenburg theory by 980 nm laser excitation at 200 mW laser power (**b**,**c**) Gain spectra by Füchtbauer–Ladenburg theory of the samples 5W and 10W at 200 mW.

The emission cross-sections are also calculated by using the Füchtbauer–Ladenburg (FL) theory, which approximates the emission cross-sections from the Er^3+^ emission spectra by the formula ${\sigma }_{ems}\left(\lambda \right)=\frac{{\lambda }^{4}}{8\pi c{n}^{2}\tau }\frac{I(\lambda )}{\int I\left(\lambda \right) d\lambda }$ where $\lambda$ is the wavelength, $I$ is the emission intensity (see Fig. 4a,b), $c$ is the vacuum velocity of light, $n$ is the refractive index and $\tau$ is the lifetime^53^. The emission cross-section spectra for the prepared samples by the FL theory are given in Fig. 7a. Namely, the ${\sigma }_{ems}\left(\lambda \right)$ can be determined from the line shape function and the intensity of the emission band “$I(\lambda )$”, as well as the radiative lifetime of the upper manifold and the refraction index. It is illustrated that no significant changes occur in the on the line shape of the Er^3+^ emission cross-sections as the WO_3_ content increases. It is important to mention that the maximum value of emission cross-section for the sample 5W obtained in Fig. 7a is 7.2 $\times$ 10^–21^ cm^2^, which is slightly less than obtained from the MC theory (7.4 $\times$ 10^–21^ cm^2^) in Fig. 5 under the same conditions. The gain spectra of the samples 5W and 10W at laser excitation power of 200 mW by the FL theory are shown in Fig. 7b,c. Similar to the emission spectra shape in Fig. 4, the gain spectra shape by the FL theory is significantly dependent on the laser power that might be attributed to the inhomogeneous broadening in which both laser power and amplifier efficiencies will be lower. Additionally, the line width of an amplifier is also affected due to the saturation only at a specific wavelength which will distort the gain spectrum^45^. Therefore, the dependence of the gain spectra shape by the FL theory on the laser power is an expected consequence of a gain system dominated by inhomogeneous broadening. The emission cross-section and the gain calculations for the samples 5W and 10W at 250 mW excitation power by the FL theory are demonstrated in Supplementary Fig. S3 of the Supplementary Material. However, the achieved values for the gain and gain bandwidth are similar to the ones reported in the literature^54,55^. Also, the FL theory shows that at least 30% of the Er^3+^ ions should be at the excited state level to obtain a gain signal in the L-band in Fig. 7b,c, which is similar to the result obtained by the MC theory when the samples are excited at 200 mW in both theories. Nevertheless, the obtained maximum gain values employing the MC theory are slightly larger, but the central peak does not show any change, i.e., a good agreement is observed between the two theories.

The MC theory is often used to predict the emission cross-section of the 1.53 μm electronic transition of Er^3+^-doped bulk glasses from the transitions of measured absorption spectrum. Furthermore, with any modification or not, it is generally employed to determine the emission cross-section in EDFAs. In this frame, FL theory is occasionally employed to predict the emission cross-section in Er^3+^-doped bulk glasses. A possible explanation for such fact is that MC theory is easy to determine since it just depends experimentally on the absorption spectrum. However, FL theory depends on the measurements of glass refractive index, emission intensity and Er^3+^ lifetime, which could result in a poor approximation of the emission cross-section unless carefully done. Besides, such measurements are uncommon in optical fibers; therefore, it is easy to apply the MC theory to determine the emission cross-section.

**Figure 8:**
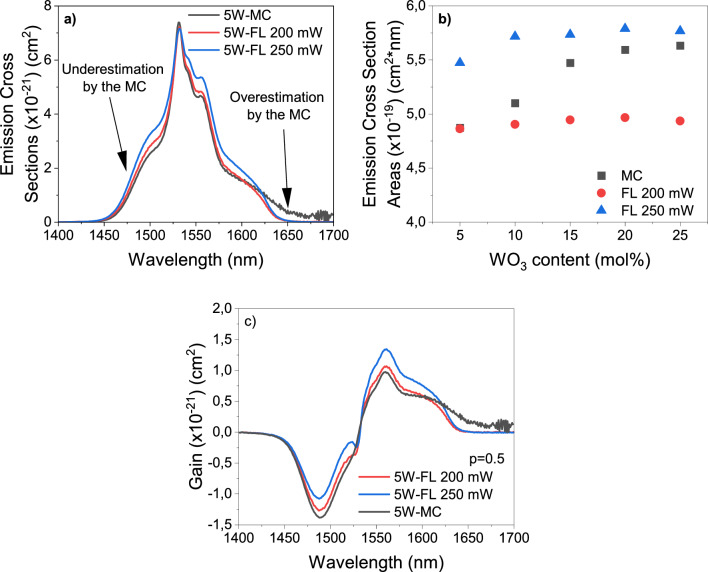
Comparison of McCumber and Füchtbauer–Ladenburg theories. (**a**) Emission cross-section spectra for 5W by 980 nm excitation (**b**) Emission cross-section areas for all samples (**c**) Gain spectra for 5W when 50% of Er^3+^ ions are at the excited state.

The comparison of the Er^3+^ emission cross-sections calculated from the MC theory and FL theory at 200 and 250 mW for the sample 5W is shown in Fig. 8a. The FL theory yields a broader spectrum than the MC theory. Such a difference can be related to the fact that MC theory employs the absorption spectra, which are measured with the beam of the transmission spectrometer lamp, i.e., without pumping power control, which can produce different absorption probabilities. Hence, these effects are not considered in the calculations of the MC theory. However, it should be noted that the peak intensities do not change at higher laser power according to the FL theory. Moreover, the MC theory results in larger emission cross-section values at longer wavelengths due to the presence of the factor that multiplies the absorption cross-section in its formula, also called reciprocity relation, which might overestimate the emission cross-section after the highest intensity peak^56^. Additionally, the areas under the emission cross-sections of all samples by both theories are compared in Fig. 8b. The emission cross-section areas increase with the WO_3_ incorporation according to the MC theory, but the FL theory suggests the emission cross-section areas experience a very little increase. Similar results are observed also in the emission spectrum in Fig. 4. Furthermore, FL theory exhibits that the emission cross-section areas increase with the laser power. Finally, Fig. 8c shows that the gain spectra calculated by both theories for 5W when there is 50% population inversion differ significantly. There is a higher gain at higher laser power by the FL theory and the MC theory yields lower gain at shorter wavelengths but higher gain at longer wavelengths.

## Conclusion

Five GeO_2_–Bi_2_O_3_–WO_3_ glasses doped with Er_2_O_3_ are prepared with varying WO_3_ content. No examples of glasses with this composition are found in the literature review. The thermal characterizations reveal that the samples with 5 and 10 mol% WO_3_ can have good thermal stabilities for fiber fabrication to be used in the L-band fiber amplifier applications. Additionally, it is shown that the Er^3+^ emission bandwidth broadens, absorption and emission cross-sections improve with the increase of tungsten atoms in the glass matrix whereas the Er^3+^ ion lifetime decreases. The emission cross-section values of the bismuth-germanate glasses in this study are similar to the tellurite glasses but larger than silica glasses reported in the literature. Moreover, the gain spectra by the MC and FL theories are calculated and 30% population inversion is found to be enough to get gain signal in the L-band. Furthermore, a comparison of both theories, which are widely used in the calculation of Er^3+^ emission cross-section, are made. The FL theory claims higher emission cross-section intensity and gain signal at shorter wavelengths, whereas the MC theory provides greater values for longer wavelengths. Also, the emission cross-section areas have similar values for all samples by the FL theory; however, they increase with the tungsten content by the MC theory. Therefore, bismuth-germanate glasses with 5 and 10 mol% of WO_3_ open new opportunities for glass photonics, which could be used in integrated optics with well-developed silicon technologies and in EDFA applications.

## Experimental methods

The used precursors for glass preparation are Germanium oxide (Adv. Materials Tech. Co., 99.999% 5N), Bismuth (III) oxide (Strem Chemicals, 99.9%-Bi), Tungsten (VI) oxide (Alfa Aesar, 99.8% (metals basis)), Antimony (III) oxide (Sigma-Aldrich, 99%), and Erbium (III) oxide (Strem Chemicals, 99.995%-Er). The precursors were melted in alumina crucibles at 1200 °C in ambient atmosphere for 45 min and cast at 1100 °C into stainless steel molds which were preheated at 460 °C. After the casting, the glasses were annealed at 460 °C for 2 h. Finally, the glasses were optically polished on both parallel sides. The fabricated samples have the composition of 97.5[(75 − x)GeO_2_–25Bi_2_O_3_–(x)WO_3_]–2Sb_2_O_3_–0.5Er_2_O_3_ where x = 5, 10, 15, 20, and 25 mol%. The labels of the samples are “5W”, “10W”, “15W”, “20W”, and “25W” concerning their WO_3_ content.

The glass with 5 mol% WO_3_ was fabricated with higher amount of erbium content up to 2 mol% Er_2_O_3_. However, the glass composition with 0.5 mol% Er_2_O_3_ concentration presents the best trade-off between glass stability and high concentration of Er_2_O_3_. Above 25 mol% of WO_3_, some crystallization spots were observed during the casting. As these glasses would be used for fiber drawing, the glass must be stable enough to be casted into a preform. Hence, we do not consider adding glasses with higher amount of WO_3_ considering the focus of this work. Sb_2_O_3_ was used to limit the reduction of bismuth and tungsten ions during the glass synthesis^57^. The glass with 5 mol% WO_3_ was fabricated with up to 8 mol% Sb_2_O_3_ in our preliminary study and the spectroscopic properties of Er^3+^ ions was not affected by higher concentration of Sb_2_O_3_. Therefore, this concentration of antimony oxide was demonstrated to be sufficient for this purpose.

The characteristic temperatures of the glasses, glass transition (T_g_) and crystallization onset (T_x_) temperatures were determined by differential scanning calorimetry (DSC) measurements, which were performed in a Pt/Rh crucible using a Netzsch DSC 404 F3 Pegasus apparatus at a heating rate of 10 °C min^−1^, from 25 to 800 °C. The glass thermal stability is determined as, $\Delta T={T}_{x}-{T}_{g}$.

Glass densities were measured by an Alfa Mirage MD-300S electronic densimeter using Archimedes’ principle. The Er^3+^ ion concentrations were calculated from: ${Er}^{3+}=M\times \rho \times x/N$ where $M$ is the molecular weight of the glass, $\rho$ is the glass density, $x$ is the Er_2_O_3_ mol% and $N$ is Avogadro’s number^58^.

The refractive indices of the glasses were measured by a prism coupling technique (Metricon 2010) at five different wavelengths 532, 633, 972, 1308 and 1538 nm with TE polarization.

The transmission spectra in the UV–Vis-NIR region were measured by Cary 60 and Cary 5000 spectrometers and in the MIR region by a Frontier FTIR spectrometer. The Raman spectra of the samples were recorded with a Renishaw in Via™ confocal Raman microscope under laser excitation at 785 nm.

The luminescence measurements were carried out with a diode laser at 980 nm with 200 and 250 mW power pumping. The luminescence light is detected with a Jobin–Yvon Nanolog spectrometer coupled with a nitrogen-cooled InGaAs detector. The Er^3+^ lifetime of the ^4^I_13/2_ → ^4^I_15/2_ radiative decay was measured by exciting the samples with an OPO at 980 nm with a frequency of 10 Hz and an average power of 30 mW per pulse. The signal was collected with an Edinburgh FLS1000 spectrometer connected to a Tektronix MDO 3022/200 MHz oscilloscope.

## Supplementary Information

Supplementary Information.

## Data Availability

The datasets generated during and/or analysed during the current study are available from the corresponding author on reasonable request.
