# Supplementary material for: The role of tungsten oxide in Er3+-doped bismuth-germanate glasses for optical amplification in L-band
Source: Sci Rep. 2023 May 31;13:8835. doi: 10.1038/s41598-023-35995-8 (PMC10232524; doi:10.1038/s41598-023-35995-8)
Supplement: Supplementary file 1 — Supplementary Information. [file 41598_2023_35995_MOESM1_ESM.docx]

**Supplementary Material**

**THE ROLE OF TUNGSTEN OXIDE IN ER^3+^-DOPED BISMUTH-GERMANATE GLASSES FOR OPTICAL AMPLIFICATION IN L-BAND**

Hüseyin Can Çamiçi*^1^, Théo Guérineau^1^, Victor Anthony Garcia Rivera^1^, Rodrigo Ferreira Falci^1^, Sophie LaRochelle^1^ and Younès Messaddeq^1^

^1^Centre for Optics, Photonics and Laser (COPL), 2375 rue de la Terrasse, Université Laval, Québec, QC, CANADA

*Corresponding author: huseyin-can.camici.1@ulaval.ca

**Sellmeier** **Coefficients**

Table S1. B_1_, B_2_, C_1_, and C_2_ Sellmeier equation coefficients of the samples

| **Sample** | **B_1_** | **B_2_** | **C_1_** | **C_2_** |
| --- | --- | --- | --- | --- |
| **5W** | 2.7374 | 8.0130 | 0.0307 | -4721.0832 |
| **10W** | 2.7666 | -22.9686 | 0.0298 | -3599.4351 |
| **15W** | 2.9578 | -23.3105 | 0.0317 | -3544.5870 |
| **20W** | 3.0107 | 23.5725 | 0.0361 | -2120.8439 |
| **25W** | 3.1431 | -15.4428 | 0.0349 | -4089.2953 |

**Linear Absorption Coefficient**

The linear absorption coefficient ($\alpha$) of the Er^3+^ ions between 1400 and 1700 nm can be found in Fig. S1. $\alpha$ is calculated from the formula: $\alpha=\frac{-1}{L}ln(\frac{T}{100})$ where $T$ is the % Transmission and $L$ is the sample thickness. The thicknesses of the glasses are 2.15, 2.60, 2.45, 2.80, and 2.75 mm with the order of increasing WO_3_. It is shown that $\alpha$ of the Er^3+^ ions are improved when the WO_3_ content increases from 5 to 25 mol%.

Fig. S1. Linear absorption coefficients of the Er^3+^ ions in the glasses

**Lifetime**

Fig. S2. Lifetime measurements of Er^3+^ ions. Inset shows the decay spectra of Er^3+^ ions. The samples are excited at 980 nm and the emission lifetime is measured at 1532 nm.

**Emission Cross-Section and Gain Calculations by Füchtbauer-Ladenburg (FL) at 250 mW laser power**

Fig. S3. a) Emission cross-sections of glasses by the Füchtbauer-Ladenburg theory by 980 nm laser excitation at 250 mW laser power b-c) Gain spectra by Füchtbauer-Ladenburg theory of the samples 5W and 10W at 250 mW
